# Les traitements du cancer de la vulve: expérience du Centre d’Oncologie d’Oujda

**DOI:** 10.11604/pamj.2018.31.182.13812

**Published:** 2018-11-15

**Authors:** Zineb Dahbi, Fouad Elmejjatti, Farid Naciri, Amine Guerouaz, Asmae Oabdelmoumen, Ali Sbai, Loubna Mezouar

**Affiliations:** 1 Service de Radiothérapie, Centre Hospitalier Universitaire d'Oujda, Oujda, Maroc

**Keywords:** Cancer, vulve, traitement, Cancer, vulva, treatment

## Abstract

Le cancer de la vulve est une affection néoplasique rare, représentant moins de 5% des cancers génitaux de la femme. L'objectif de ce travail est de décrire le profil épidémiologique, clinique, paraclinique, thérapeutique et évolutif du cancer de la vulve chez la population de la région de l'Oriental du Maroc, ceci à travers une analyse rétrospective de toutes les patientes suivies pour un cancer de la vulve, de juin 2007 à janvier 2014, et traitées au sein de l'hôpital d'Oncologie du Centre Hospitalier Universitaire Mohamed VI de Oujda au Maroc. Notre analyse rétrospective a porté sur 34 patientes, d'une médiane d'âge de 65,7 ans, dont 52,9% étaient des multipares. Le motif de consultation dominant était le prurit dans 94.1% des cas. La moyenne du délai de consultation était de 16 mois, allant de 2 mois à 8 ans. L'ignorance et la pudeur ont été les causes majeures de ce retard diagnostique, puisque 73.5% des patientes avaient déjà une maladie localement avancée au diagnostic. Le traitement chirurgical a été proposé à 61.4% des cas, il a consisté en une vulvectomie radicale avec un curage inguinal bilatéral dans 68.5% des cas. Le recours à la radiothérapie adjuvante a été indiqué chez 41.2% des cas, 5.9% des patientes ont bénéficié d'une radiothérapie néo adjuvante, et 20,6% d'une radiothérapie exclusive associée à une chimiothérapie concomitante. La chimiothérapie palliative a été proposée pour 8.8% des patientes. Le taux de survie globale à 3 ans est à 65%, le taux de récidives locorégionales ou à distance est de 17.3% des cas. Les particularités culturelles et sociales des patientes de la région de l'Oriental du Maroc, qui sont suivies pour un cancer de la vulve, sont des facteurs influençant le traitement et ses résultats. Des efforts de prévention et de sensibilisation supplémentaires sont à mener afin de réduire l'incidence des stades localement avancés, et de permettre un traitement curatif à cette population.

## Abstract (en)

Vulvar cancer is a rare neoplastic disorder accounting for less than 5% of female genital cancers. This study aims to describe the epidemiological, clinical, paraclinical, therapeutic and evolutionary profile of vulvar cancer in the population of the eastern region of Morocco. We conducted a retrospective study of all patients treated for vulvar cancer in the Oncology Center, University Hospital Mohammed VI in Oujda, Morocco from June 2007 to January 2014. The study included 34 patients with an average age of 65.7 years, of whom 52.9% were multiparous. The most frequent reason for consultation was pruritus (94.1% of cases). The median of consultation time was 16 months, ranging from 2 months to 8 years. Ignorance and modesty were the major causes of diagnostic delay, since 73.5% of patients already had locally advanced disease at diagnosis. Surgical treatment was proposed to 61.4% of cases and was based on radical vulvectomy associated with bilateral inguinal lymphadenectomy in 68.5% of cases. Adjuvant radiotherapy was indicated in 41.2% of cases, 5.9% of patients underwent neo-adjuvant radiotherapy while 20.6% underwent exclusive radiotherapy associated with concomitant chemotherapy. Palliative chemotherapy was proposed to 8.8% of patients. The overall survival rate at 3 years was 65%, locoregional or distant recurrence rate was 17.3%. Cultural and social characteristics of patients of the eastern region of Morocco treated for vulvar cancer are factors influencing treatment and outcomes. Additional prevention and awareness efforts should be made in order to reduce the rates of locally advanced tumors and to enable curative treatment for this population.

## Introduction

Le cancer de la vulve constitue 5% des cancers gynécologiques, qui touchent essentiellement la femme âgée. Le type histologique épidermoïde représente 90% de l'ensemble de ces cancers, ou le *human papilloma virus* (HPV) joue un rôle capital dans la carcinogenèse. La richesse des modes de manifestation de ce cancer contraste avec le retard diagnostic chez la plupart des patientes de la région de l'Oriental du Maroc, où la pudeur, souvent, les conduit à consulter à un stade avancé, où les options thérapeutiques sont moindres. L'objectif est de décrire le profil épidémiologique, clinique, histologique, thérapeutique et évolutif des cancers de la vulve, chez la population orientale.

## Méthodes

Il s'agit d'une étude rétrospective, menée au Service de Radiothérapie de l'hôpital d'Oncologie du Centre Hospitalier Universitaire Mohamed VI d’Oujda, concernant une série de 35 patientes suivies pour un cancer vulvaire, sur une période de 7 ans de juin 2007 à janvier 2014. L'ensemble des données épidémiologiques, cliniques, histologiques, thérapeutiques et évolutives a été recueilli des dossiers de l'archive de l'hôpital, et a été analysé via le logiciel SPSS, afin de comparer les résultats de chaque traitement.

## Résultats

**Figure 1:**
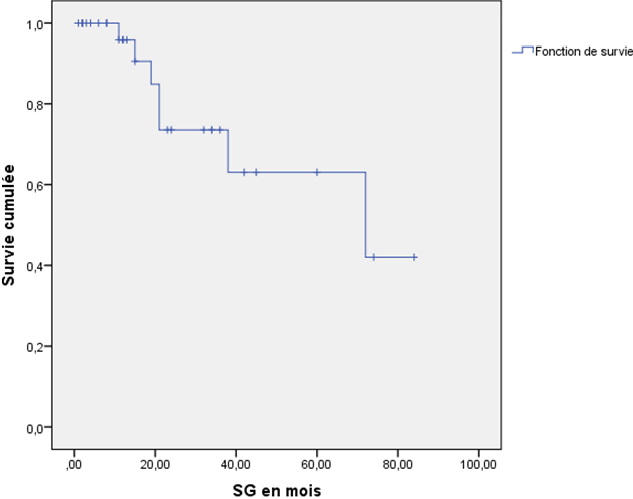
courbe de survie globale à 3 ans selon Kaplan-Meier

**Figure 2:**
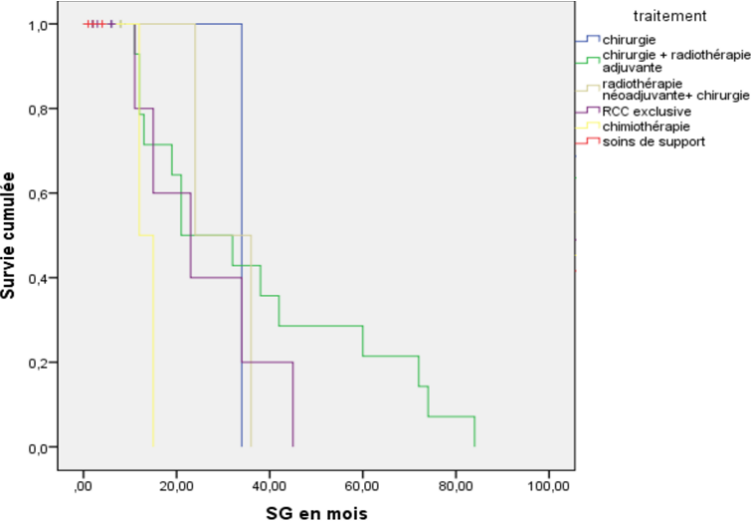
courbe de survie globale selon Kaplan-Meier stratifiée en fonction du traitement

Nous avons recensé 34 patientes, dont l'âge moyen était 65,7 ans (extrêmes 25-94 ans), elles étaient des multipares dans 52,9% des cas, et ménopausées dans 82,3% des cas. 26,4% des patientes avaient des comorbidités. 7,6% des patientes ont présenté des lésions précancéreuses. Le délai moyen de consultation était de 16 mois (2 mois - 8 ans). Les signes cliniques étaient dominés par le prurit dans 94,1% des cas, une masse vulvaire révélatrice dans 61,3% des cas, les douleurs pelviennes et des leucorrhées dans 29.4% et 82.3% des cas, respectivement. Il s'agissait de lésions multifocales dans 88,3% des cas, et d'aspect ulcéro-bourgeonnant dans 62,8% des cas (Tableau 1). La biopsie vulvaire était réalisée pour toutes les patientes, elle a révélé un carcinome épidermoïde dans 98% des cas, en guise d'un bilan d'extension locorégional et à distance, une IRM pelvienne a été réalisée pour 42% des patients, une TDM thoraco-abdominale pour 76 % des patientes, une endoscopie digestive basse ou de l'arbre urinaire a été réalisée chez 17% des patientes (Tableau 2). Au terme du bilan d'extension, les tumeurs étaient classées stade I chez 2.9% des patientes, stade II chez 23,5%, de stade III chez 14,7% et de stade IV chez 58,8% des patientes. L'attitude thérapeutique curative a été adoptée chez 82.3% des patientes, une chirurgie radicale a été réalisée chez 61,4% des patientes, une vulvectomie partielle a été proposée pour 17% des patientes. Le curage inguinal bilatéral a été réalisé chez 62% des cas. La médiane des délais entre la consultation et la chirurgie était de 8 semaines. La radiothérapie était indiquée chez 67.6% des patientes, elle était préopératoire chez 5,9% des cas, 41,2% des cas en postopératoire. La médiane des délais entre la chirurgie et la radiothérapie adjuvante était de 4 semaines. Une radiothérapie exclusive a été délivrée chez 20,6% des cas. Aucune patiente n'a bénéficié d'une curiethérapie. Une chimiothérapie palliative a été proposée pour 8,8% des patientes (Tableau 3). Après une durée moyenne de suivi de 50 mois (24 mois-81 mois), La médiane de survie globale à trois ans est de 65% (Figure 1), nous avons observé 17,3% de récidive locorégionale et à distance, dont 8.5% ont bénéficié d'une chirurgie de rattrapage, 2,8% d'une radiothérapie, et 11,4% d'une chimiothérapie palliative (Figure 2).

**Tableau 1:** paramètres épidémiologiques des patientes de la série

Paramètres	Variables	Nombre	Pourcentage (%)
**Age (année)**	>40	11	32,3
	<40	23	67,6
**Statut ménopausique**	Ménopausée	28	82,3
	Non-ménopausée	6	17,6
**Parité**	Nullipare	5	14,7
	Paucipare	11	32,3
	multipare	18	52,9
**Lésion pré-cancéreuse**		6	17,6
**Comorbidités**	Diabète	2	5,8
	HTA	3	8,88
	Obésité	3	8,88
	Autres cancers	1	2,9

**Tableau 2:** paramètres cliniques et radiologiques des patientes de la série

Paramètres	Variables	Nombre	Pourcentage (%)
**Signes cliniques**			
	Prurit	32	94,1
	Masse vulvaire	21	61,3
	Douleurs périnéales	10	29,4
	Ecoulement vaginal	28	82,3
**Extension locorégionale**			
	**focalité**		
	Unifocale	4	11,7
	Multifocale	30	88,3
	**Extension tumorale**		
	Localisé	1	2,9
	Etendu: bas Urètre/vagin/anus	8	23,5
	Etendu: haut urètre/Vessie/rectum/pelvis	5	14,7
	**Extension ganglionnaire**		
	Absence d’adenopathies	14	41,1
	Adenopathies inguinales Unilaterales	11	32,3
	Adenopathies inguinales bilaterales	9	26,4
**Extension à distance**			
	Métastases pulmonaires	1	2,9
	Métastases hépatiques	2	5,8
	Autres	0	-
**Stade**			
	I	1	2,9
	II	8	23,5
	III	5	14,7
	IV	20	58,8

**Tableau 3:** modalités thérapeutiques entreprises pour les patients de la série

Variables	Nombre	Pourcentage (%)
Chirurgie	5	14,7
Chirurgie+ radiothérapie adjuvante	14	41,2
Radiothérapie néo adjuvante+ chirurgie	2	5,9
Radio chimiothérapie concomitante exclusive	7	20,6
Chimiothérapie palliative	3	8,8
Soins de support	3	8,8

## Discussion

Le cancer de la vulve représente 5% des cancers gynécologiques [1]. Son incidence augmente avec l'âge, avec une tendance mondiale à survenir chez des femmes de plus en plus jeunes [2]. L’une de nos patientes a présenté un carcinome épidermoïde de la vulve à l'âge de 25 ans, l'âge précoce de ses premiers rapports sexuels ainsi que l'absence de vaccination sont des facteurs de carcinogenèse potentiels [3]. Le délai d'apparition des premiers symptômes et la consultation ont été étudiés dans notre série, ils ont été identifiés comme un facteur pronostique dont dépend la survie globale. Sur une série rétrospective publiée par l'équipe de l'Institut National d'Oncologie au Maroc, ce délai était de 7 mois, versus 16 mois pour nos patientes [4], un retard de consultations qui explique la fréquence des stades localement avancés au sein de notre structure, pour des raisons d'ignorance et surtout de pudeur de la population de la région de l'Oriental du Maroc. Le traitement principal du cancer de la vulve est chirurgical, il repose sur la vulvectomie totale associée à un curage inguinal bilatéral avec incisions séparées ; c’est le traitement standard des cancers vulvaires localisés. Il a été pratiqué chez 68.2% des cas. La qualité de cette résection ainsi que l'envahissement ganglionnaire inguinale sont des facteurs pronostiques majeurs justifiant ce traitement. La vulvectomie partielle peut être indiquée dans les stades précoces, la place du ganglion sentinel dans le traitement chirurgical du cancer de la vulve est en cours d'évaluation. Le lâchage de suture et le lymphœdèmes constituent les principales complications du traitement chirurgical. Le taux de ces complications post-opératoires observées chez les patientes de notre série est similaire à celui décrit dans la littérature [5].

Le cancer épidermoïde de la vulve est considéré comme un cancer radiosensible, l'irradiation peut se discuter en néo adjuvant chez les patientes porteuses de tumeurs localement avancés (> T2,N+) pour permettre un down-staging tumoral et une chirurgie d'exérèses carcinologiques par la suite, la radiothérapie adjuvante par contre est considérée comme un standard thérapeutique en cas d'envahissement ganglionnaire, en cas de limites tumorales ou de tumeur large de plus de 4cm, ou en cas de présence d'embols vasculaires ou d'engainement péri-nerveux, après une chirurgie d'exérèse. La radiothérapie exclusive peut être proposée chez les patientes récusées chirurgicalement pour des raisons médicales, en cas de tumeurs très localement avancées jugées irrésécables, ou en cas de récidive tumorale locale [6, 7]. La curiethérapie est d'indication rare, c'est surtout dans le traitement des reliquats tumoraux au contact de l'urètre ou dans le tiers inférieur du vagin ou lors des traitements conservateurs des tumeurs de moins de 3cm. La radiothérapie était indiquée chez 67.6% des patientes, elle était préopératoire chez 5.9% des cas, 41.2% des cas en postopératoire, la médiane des délais entre la chirurgie et la radiothérapie adjuvante était de 4 semaines. Une radiothérapie exclusive a été délivrée chez 20.6% des cas. Aucune patiente n'a bénéficié d'une curiethérapie.

L'irradiation externe pelvienne est délivrée le plus souvent par une combinaison de photons et d'électrons au niveau de la vulve, des creux inguinaux et parfois des chaînes iliaques, en pré et postopératoire à une dose de 45 à 50 Gy délivrés en 5 à 6 semaines. En cas de radiothérapie exclusive, les doses délivrées sont comprises entre 60 et 70 Gy dans le volume vulvaire et entre 55 et 60 Gy dans les volumes inguino-cruraux. La radiothérapie est réalisée dans notre série d'une façon exclusive dans 7,4% des cas le plus souvent à titre palliatif, en préopératoire dans 5,7% des cas dans les formes localement avancées inaccessibles à un traitement chirurgical d'emblée, en postopératoire dans 37.2% des cas. La radiothérapie postopératoire est pratiquée devant des facteurs de risque de récidive locale: un volume tumoral supérieur à 4cm; une marge chirurgicale étroite (moins de 8 mm), un envahissement vasculaire lymphatique profond et le statut ganglionnaire positif. Pour la radiothérapie néo adjuvante ou exclusive, indiquée devant les formes localement avancées, ou récidivantes, l'association avec une chimiothérapie concomitante à base de 5 fluroracile ou de cisplatine semble améliorer les taux de résécabilité et avec un taux de réponse complète à 40% chez quelques patientes rapportées sur des séries rétrospectives. En adjuvant, la chimio-concomitante associée à la radiothérapie après traitement chirurgical n'a pas encore été évaluée. Aucune patiente de la série n'en a bénéficié [8]. Tous stades confondus, une corrélation statistiquement significative a été retrouvée entre le traitement proposé et la survie globale, en faveur de l'association radiothérapie adjuvante et chirurgie radicale, qui semblent être supérieures à la radio chimiothérapie concomitante exclusive qui est également supérieure à la chirurgie seule. Un résultat qui se concorde globalement avec les résultats de la littérature [4, 9]. L'utilisation de la chimiothérapie en tant que traitement palliatif a été proposée pour 8,8% des patientes de notre série, en littérature, son usage demeure encore peu étudié et les résultats disponibles sont décevants. Les protocoles les plus utilisés sont l'association 5FU, cisplatine, et bléomycine méthotrexate. Le paclitaxel, et le geftinib sont en cours d'évaluation dans cette indication. Le taux de survie globale des patientes traitées pour un cancer de la vulve, tous stades confondus, est de 57 à 62,5%, ce qui se rapproche aux taux décrit pour notre série [10, 11].

## Conclusion

Les particularités culturelles et sociales des patientes de la région de l'Oriental du Maroc, qui sont suivies pour un cancer de la vulve, sont des facteurs influençant le traitement et ses résultats. Des efforts de prévention et de sensibilisation supplémentaires sont à mener afin de réduire l'incidence des stades localement avancées, et de permettre un traitement curatif à cette population.

### Etat des connaissances actuelles sur le sujet

- Cancer de la vulve est désormais le cancer gynécologique qui a bénéficié le moins des avancées thérapeutiques qu'a connu le monde de cancérologie moderne;
- Du fait de sa rareté, la littérature compte peu de publications la concernant, il s'agit surtout de revues rétrospectives.

### Contribution de notre étude à la connaissance

- Notre travail constitue un penchant sur les thérapies actuelles proposées, par notre institution, pour les patientes porteuses d'un cancer de la vulve, ainsi que leurs résultats en termes de survie.

## Conflits des intérêts

Les auteurs ne déclarent aucun conflit d'intérêts.
